# Prophylactic immune priming with heat-killed *Lacticaseibacillus rhamnosus* combined with therapeutic *Lactiplantibacillus plantarum* cell-free supernatant protects against *Pseudomonas aeruginosa* lung infection in mice

**DOI:** 10.3389/fimmu.2026.1802599

**Published:** 2026-04-01

**Authors:** Weichen Gong, Luciano Arellano-Arriagada, Leonardo Albarracin, Julio Nicolás Argañaraz Aybar, Ayelen Baillo, Solange Cisterna-Vergara, Keita Nishiyama, Juan Carlos Valdéz, Nadia Gobbato, Haruki Kitazawa, Julio Villena

**Affiliations:** 1 Food and Feed Immunology Group, Laboratory of Animal Food Function, Graduate School of Agricultural Science, Tohoku University, Sendai, Japan; 2 Livestock Immunology Unit, International Education and Research Centre for Food and Agricultural Immunology (CFAI), Graduate School of Agricultural Science, Tohoku University, Sendai, Japan; 3 Laboratory of Respiratory Immunology (LaRI), Division of Animal Immunology and Omics, International Education and Research Center for Food and Agricultural Immunology (CFAI), Graduate School of Agricultural Science, Tohoku University, Sendai, Japan; 4 Laboratory of Immunology, Faculty of Biochemistry, Chemistry and Pharmacy, National University of Tucuman, Tucuman, Argentina; 5 Laboratory of Immunobiotechnology, Reference Centre for Lactobacilli (CERELA-CONICET), Tucuman, Argentina

**Keywords:** alveolar macrophages, *cell-free culture supernatant*, Lacticaseibacillus rhamnosus CRL1505, Lactiplantibacillus plantarum ATCC10241, postbiotics, *Pseudomonas aeruginosa*, respiratory infection

## Abstract

**Background:**

*Pseudomonas aeruginosa* is a leading cause of severe respiratory infections in immunocompromised individuals, and its increasing antibiotic resistance poses a major global health challenge. Antibiotic-resistant strains exhibit enhanced biofilm-forming capacity, contributing to persistent infection, immune evasion, and poor clinical outcomes. Innovative non-antibiotic strategies that simultaneously enhance host immunity and directly target bacterial virulence are urgently needed.

**Methods:**

In this study, we evaluated a dual non-viable microbial therapeutic strategy combining prophylactic immune priming with heat-killed *Lacticaseibacillus rhamnosus* CRL1505 (HK1505) and therapeutic aerosol administration of *Lactiplantibacillus plantarum* ATCC^®^ 10241™ cell-free culture supernatant (LpCFS) in murine models of lung infection caused by antibiotic-sensitive (PaS) and multidrug-resistant (PaR) *P. aeruginosa*. Pulmonary bacterial burden, systemic dissemination, and lung injury markers were assessed. In addition, cytokine/chemokine profiles were evaluated in the respiratory tract *in vivo* and in primary cultures of broncho-alveolar lavage (BAL) macrophage-enriched adherent cells *ex vivo*. HK1505 and LpCFS treatments were evaluated separately and in combination.

**Results:**

LpCFS did not directly alter cytokine or chemokine production by BAL macrophage-enriched adherent cells, indicating the absence of intrinsic immunostimulatory activity. However, therapeutic aerosol administration of LpCFS significantly reduced pulmonary and systemic PaS and PaR loads, attenuated lung damage, and modulated the inflammatory response by decreasing pro-inflammatory cytokines while increasing IL-10 during infections. Prophylactic administration of HK1505 effectively primed BAL macrophage-enriched adherent cells, enhancing their production of IL-1β, IL-6, IFN-γ, and IL-27 while reducing TNF-α and chemokine expression (CCL2, CXCL2, and CXCL10), thereby promoting efficient bacterial clearance with limited immunopathology. In this set of experiments HK1505 was compared with the live *L. rhamnosus* CRL1505 and notably, HK1505 retained the immunostimulatory efficacy of the viable bacteria. In addition, the combination of the prophylactic HK1505 administration and the therapeutic LpCFS treatment provided superior protection against the respiratory infections than individual treatments. The combined approach completely prevented lung infection and bacteremia in PaS-infected mice and significantly improved the outcomes in PaR infection.

**Conclusion:**

Our findings demonstrate that a non-viable probiotic-based strategy integrating prophylactic immune priming with HK1505 and therapeutic antibiofilm intervention with LpCFS effectively protects against antibiotic-resistant *P. aeruginosa* lung infection. This approach highlights the translational potential of postbiotic immunomodulation as a safe and innovative alternative for managing multidrug-resistant respiratory infections.

## Introduction

*Pseudomonas aeruginosa* is a major opportunistic pathogen responsible for severe respiratory infections, particularly in immunocompromised individuals, patients with chronic lung diseases, and those receiving mechanical ventilation (1). The clinical management of *P. aeruginosa* infections has become increasingly challenging due to the rapid global spread of antibiotic-resistant strains. Multidrug-resistant *P. aeruginosa* is associated with prolonged hospitalization, high mortality rates, and limited therapeutic options, representing a critical threat to public health (2). A key feature underlying the persistence and antibiotic tolerance of resistant strains is their enhanced ability to form biofilms, which protect bacteria from antimicrobial agents and host immune responses, facilitating long-term colonization of the respiratory tract (3, 4).

Traditional antibiotic-based therapies often fail to eradicate biofilm-associated *P. aeruginosa*, particularly in the context of multidrug resistance. Moreover, excessive or uncontrolled host inflammatory responses can further exacerbate lung injury, contributing to poor outcomes even when bacterial burdens are partially reduced (3, 4). Therefore, effective control of *P. aeruginosa* lung infection requires not only direct antibacterial activity but also precise modulation of host immune responses to achieve efficient pathogen clearance while limiting immunopathology. This has prompted increasing interest in alternative or complementary therapeutic strategies that target both bacterial virulence and host immunity without relying solely on conventional antibiotics.

Immunobiotics and postbiotics have emerged as promising candidates for modulating respiratory immunity (5). Among them, *Lacticaseibacillus rhamnosus* CRL1505 has been extensively characterized as a potent immunomodulatory strain capable of enhancing resistance to respiratory pathogens (6). Previous studies have demonstrated that the nasal administration of the CRL1505 strain to mice enhances pulmonary innate immune responses primarily through the activation of alveolar macrophages (6). In addition, this immunobiotic bacterium is able to beneficially modulate the Toll-like receptor 4 (TLR4) signaling in macrophages (7). Through the modulation of alveolar macrophages, the CRL1505 strain modulates the balance of inflammatory and anti-inflammatory cytokines and chemokines in the respiratory tract, resulting in improved resistance to both viral and bacterial respiratory infections, including complex scenarios such as viral–bacterial superinfection. Importantly, accumulating evidence indicates that the immunomodulatory effects of nasally administered CRL1505 are largely preserved after heat inactivation, suggesting that bacterial viability is not required for immune priming (8). This characteristic makes heat-killed *L. rhamnosus* CRL1505 (HK1505) particularly attractive as a prophylactic immunomodulatory strategy with an improved safety profile for clinical application.

In contrast to immune-priming approaches, direct targeting of *P. aeruginosa* virulence factors, especially biofilm formation, represents a critical therapeutic need during active infection. Cell-free supernatants derived from lactic acid bacteria have been shown to exert antimicrobial and antibiofilm activities independent of live bacteria. Notably, the cell-free supernatant from *Lactiplantibacillus plantarum* ATCC^®^ 10241™ (LpCFS) has been reported to effectively inhibit biofilm formation and directly reduce the viability of *P. aeruginosa*, including antibiotic-resistant and antibiotic-sensitive strains (9, 10). Furthermore, it was demonstrated recently that in mice infected with antibiotic-sensitive (PaS) or multidrug-resistant (PaR) *P. aeruginosa* strains isolated from cystic fibrosis (CF) patients, the nebulization with the LpCFS reduced the severity of the respiratory infection in terms of bacterial burden as well as detrimental inflammation (10). These properties suggest that LpCFS may serve as a therapeutic agent capable of weakening bacterial defenses and facilitating pathogen clearance without inducing excessive host inflammation.

Based on these backgrounds, we hypothesized that combining prophylactic immune priming with HK1505 and therapeutic administration of LpCFS would provide synergistic protection against *P. aeruginosa* lung infection. In this study, we evaluated this dual non-viable microbial strategy in murine models of *P. aeruginosa* respiratory infections using PaS or PaR strains previously isolated from CF patients (10). We investigated the effects of HK1505 and LpCFS, alone or in combination, on pulmonary and systemic bacterial burden, lung epithelial injury, and cytokine and chemokine responses. We also investigated the effect of these treatments specifically in the profile of cytokines induced by broncho-alveolar lavage (BAL) macrophage-enriched adherent cells constituted mainly by resident alveolar macrophages. Our *in vivo* and *ex vivo* results reveal that integrating immune priming with the postbiotic HK1505 and pathogen-directed therapy with LpCFS offers an effective and safe approach to controlling *P. aeruginosa* respiratory infection, highlighting the translational potential of this dual non-viable microbial strategy.

## Methods

### Bacterial strains and microbial preparations

*Lacticaseibacillus rhamnosus* CRL1505 was obtained from the CERELA-CONICET culture collection (Chacabuco 145, San Miguel de Tucumán, Argentina). All lactobacilli cultures were kept freeze-dried. Lactobacilli were cultured for 12 h at 37 °C (final log phase) in Man-Rogosa-Sharpe broth (MRS, Oxoid). The bacteria were harvested, washed three times phosphate buffer saline (PBS, pH 7.2), and resuspended in sterile PBS. Non-viable *L. rhamnosus* CRL1505 (HK1505) was obtained by tyndallization in a water bath at 80 °C for 30 min. *L. plantarum* ATCC^®^ 10241™ strain was cultured in MRS broth (Britania, Argentina) at 37 °C. The supernatant from lactobacilli culture (LpCFS) was obtained as described previously (10) and conserved at 4 °C.

Strains of antibiotic-sensitive *P. aeruginosa* (strain PaS), and multi-resistant *P. aeruginosa* (strain PaR) were isolated from sputum samples of CF patients using standard clinical microbiological methods and tested to infect mice as described previously (10). The PaS strain is sensitive to ceftazidime, ciprofloxacin, gentamicin, amikacin, imipenem, meropenem, piperacillin/tazobactam and colistine while PaR is resistant to all the mentioned antibiotics except for colistin (10). In addition, it was previously demonstrated that the PaR strain displayed greater biofilm-forming capacity than the PaS strain (10).

### Animal model of lung infection and treatments

Six-week-old male BALB/c mice from a random-bred colony maintained at the Institute of Microbiology, National University of Tucumán (Argentina), were randomly assigned to experimental groups. Animals were housed under standard conditions with *ad libitum* access to food and water and a 12 h light/dark cycle. For infection or euthanasia procedures, mice were anesthetized by intraperitoneal administration of ketamine hydrochloride (75 mg/kg) and medetomidine hydrochloride (1 mg/kg). The Institutional Animal Care and Use Committee at the University of Maryland, Baltimore, MD, approved the experiments and it was endorsed by the Bioethics Committee of the Faculty of Biochemistry, Chemistry and Pharmacy, National University of Tucuman (Tucuman, Argentina).

A model of lung infection with *P. aeruginosa* using BALB/c mice previously described was used (10). Briefly, animals were nasally challenged with 50 μL of PaS, or PaR suspensions (10^6^ CFU), for two consecutive days.

**Figure 1:**
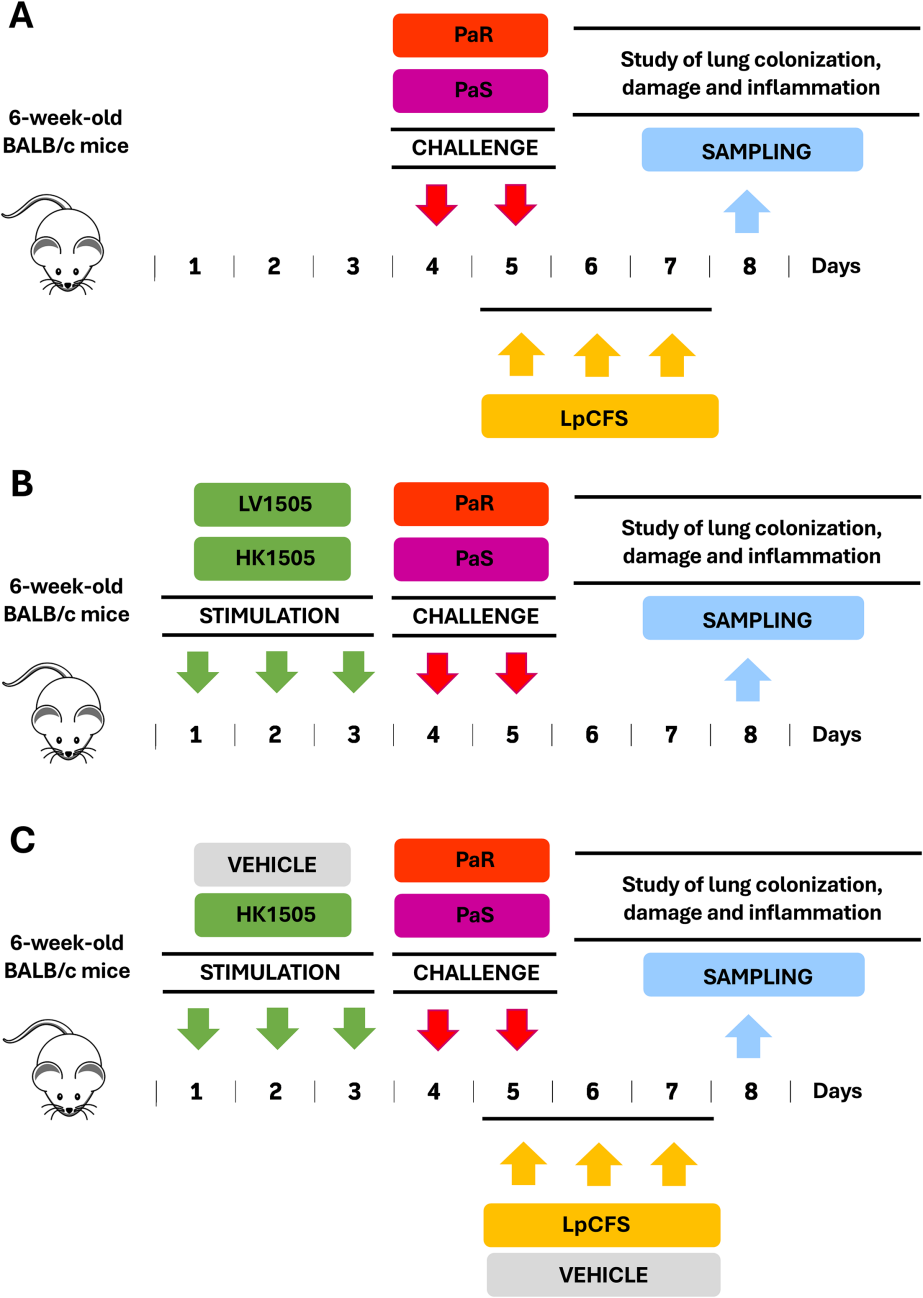
Experimental workflows used in this work. **(A)** Therapeutic administration of *Lactiplantibacillus plantarum* ATCC 10241 cell-free supernatant (LpCFS). BALB/c mice (6-week-old) were nasally challenged with antibiotic-sensitive (PaS) or multidrug-resistant (PaR) *Pseudomonas aeruginosa* strains for two days and then received aerosol administration of LpCFS for three consecutive days after infection. **(B)** Prophylactic immune priming with live (LV1505) and heat-killed (HK1505) *Lacticaseibacillus rhamnosus* CRL1505. BALB/c mice (6-week-old) were nasally treated with LV1505, or HK1505 for three consecutive days and then infected with PaR or PaS for two days. **(C)** Prophylactic immune priming with HK1505 combined with therapeutic LpCFS. BALB/c mice (6-week-old) were nasally treated with LV1505, HK1505, or vehicle control for three consecutive days and then infected with PaR or PaS for two days. In addition, mice received aerosol administration of LpCFS or vehicle control for three consecutive days after infection. In the three sets of experiments, bacterial counts in lung and blood, lung injury markers, and cytokine profiles in the respiratory tract were evaluated in the time point indicated as “sampling”. Data of the same vehicle control cohort was used for all the comparisons.

For the study of the effect of the therapeutic administration of LpCFS on the resistance to *P. aeruginosa* respiratory infection, mice were challenged with PaS or PaR strains and then received aerosol administration of LpCFS for three consecutive days (**Figure 1A**). Nebulizations with LpCFS were performed for 5 minutes by day. The nebulization was conducted by a compressor/nebulizer (OMROM NE-C801) connected by a tube to a hermetically sealed plastic container as described before (10). The mice to be nebulized were placed into this container.

For the evaluation of the effect of the prophylactic administration live (LV1505) and heat-killed (HK1505) *L. rhamnosus* CRL1505 on the resistance to *P. aeruginosa* respiratory infection, mice received prophylactic intranasal administration of LV1505 or HK1505 for three days (10^8^ cells/mouse/day) and then were challenged with PaS or PaR strains as described above (**Figure 1B**).

In addition, both the prophylactic administration of LV1505 and HK1505 in combination with the therapeutic administration of LpCFS was assessed (**Figure 1C**). A group of control mice received intranasal administration of PBS for three days, were infected with PaS or PaR, and then treated with aerosol administration of PBS for three consecutive days (**Figure 1C**). This group was designated as vehicle control and was used as a control cohort for all the comparisons.

The administration routes and doses of LpCFS, LV1505 and HK1505 used in this study were selected based on previous studies demonstrating their efficacy in inducing beneficial effects in the respiratory tract (6, 10).

In the three sets of experiments, three days after the PaS or PaR infections, the lung and blood bacterial cell counts, and the levels of injury markers and cytokines in broncho-alveolar fluid (BAL) samples were determined (**Figure 1**). Body weight was expressed as a percentage of the initial weight (day 0 = 100%).

### Quantitative lung and blood bacteriology

To quantify bacterial loads in the respiratory tract, whole lungs were aseptically removed, weighed, and homogenized in 5 mL of sterile 0.9% saline solution. In addition, blood samples were taken aseptically. Serial dilutions of the lung homogenates and blood samples were immediately plated onto Levin, and BCSA agar (BioMérieux^®^, Argentina). Viable bacterial counts were determined after overnight incubation at 37 °C.

### Studies in BAL

BAL samples were collected as described before (6). Briefly, 0.5 mL of saline solution containing 10 U/mL of heparin was injected and aspirated through the tracheostomy tube three times. The total volume of BAL fluid was pooled. The determination of elastase activity was carried out following the procedures described previously (10). Briefly, 500 µL of elastin-congo red solution (20 mg of congo red elastin in 1 mL of 10 mM Na_2_HPO_4_, pH=7) was used as a substrate. Samples (500 µL of BAL) were incubated overnight with shaking for 24 h at 37°C. The pellet was removed by centrifugation, and the absorbance of the supernatant was read in a spectrophotometer at OD495 nm. Albumin content, a measure to quantitate increased permeability of the bronchoalveolar–capillarity barrier, and lactate dehydrogenase (LDH) activity, an indicator of general cytotoxicity, were determined in the acellular BAL fluid as described previously (6).

Interferon (IFN)-β (Mouse IFN-beta ELISA Kit), IFN-γ (Mouse IFN-gamma Quantikine ELISA Kit), TNF-α (Mouse TNF alpha ELISA Kit, High Sensitivity, BMS607-2HS), IL-6 (Mouse IL-6 ELISA Kit, BMS603-2), IL-1β (Mouse IL-1 beta ELISA Kit, BMS6002), IL-27 (Mouse IL-27 p28/IL-30 ELISA Kit Quantikine, M2728), CCL-2(Mouse CCL2/JE/MCP-1 ELISA Kit Quantikine, MJE00B), and IL-10 (Mouse IL-10 Quantikine ELISA Kit) concentrations in BAL samples were measured with commercially available enzyme-linked immunosorbent assay (ELISA) technique kits following the manufacturer’s recommendations (R&D Systems, MN, USA). In addition, CXCL2 (Mouse MIP-2/CXCL2 ELISA Kit) and CXCL10 (Invitrogen™ Mouse IP-10 (CXCL10) ELISA Kit) from ThermoFisher Scientific (Waltham, MA,USA) were used.

### BAL macrophage-enriched adherent cells primary cultures

Primary cultures of murine BAL macrophage-enriched adherent cells were performed as described previously (6). Briefly, BAL cells were obtained from infant mice via BAL samples and transferred to new sterile tubes, washed twice in sterile PBS, and resuspended in supplemented RPMI 1640 medium. BAL cells were seeded in 24-well plates and incubated to promote adherence. Non-adherent cells were washed, and BAL macrophage-enriched adherent cells were maintained for 24 hours before stimulation. Of note, the resulting adherent cell population is widely considered to be enriched in alveolar macrophages, although the presence of other recruited myeloid cells cannot be completely excluded, particularly under inflammatory conditions.

For challenge experiments, BAL macrophage-enriched cells were stimulated with PaS (MOI = 10), PaR (MOI = 10), and LPS (100 ng/mL). One day after the challenges, the levels of TNF-α, IL-1β, IL-6, CCL2, CXCL2, CXCL10, IFN-γ, IL-10, and IL-27 were measured in culture supernatants using ELISA.

### Statistical analysis

In all *in vivo* experiments, six mice were included per experimental group (n = 6), and each mouse was considered an independent biological replicate. Because the same lung cannot be used simultaneously for bacteriology and BAL studies, independent cohorts of mice were used for each type of analysis. In the case of ELISA measurements, samples from each mouse were analyzed in technical triplicates to ensure measurement accuracy, and in the figures the dots show the average for each mouse. The results were expressed as mean values with standard deviations. Statistical analyses were performed using GraphPad Prism 8 software (La Jolla, CA, USA). Unpaired Student’s t-test (for pairwise comparisons of the means) or one-way analysis of variance (ANOVA), followed by Tukey’s multiple-comparison test (for the comparison of multiple groups) were used to test for differences between the groups. Differences were considered significant at a p value < 0.05.

## Results

### LpCFS does not directly modulate BAL macrophage-enriched adherent cells cytokine responses

**Figure 2:**
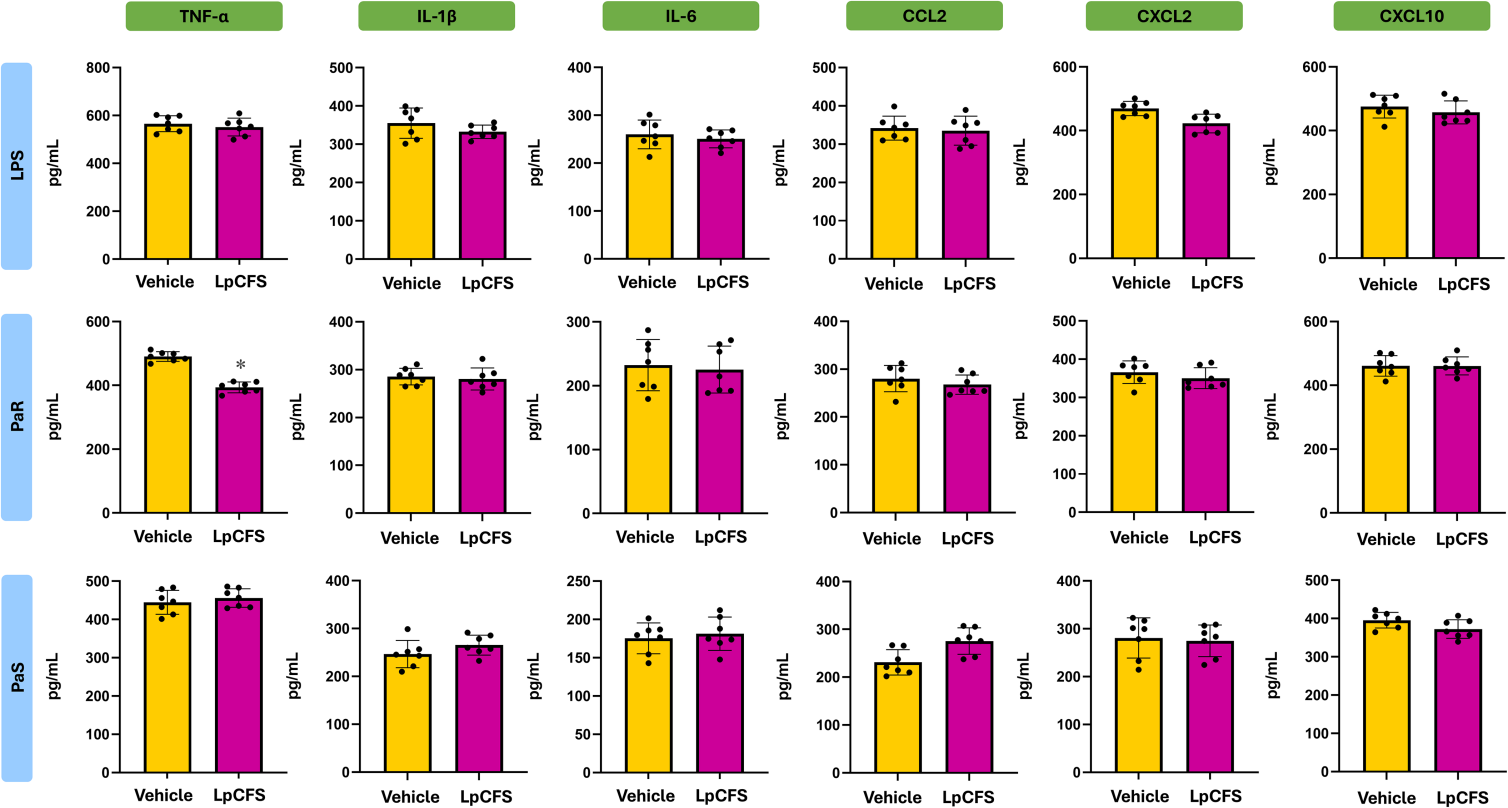
Effect of the administration of *Lactiplantibacillus plantarum* ATCC 10241 cell-free supernatant (LpCFS) on broncho-alveolar fluid (BAL) macrophage-enriched adherent cells. BALB/c mice (6-week-old) received aerosol administration of LpCFS or vehicle control for three consecutive days. On day four, BAL macrophage-enriched adherent cells were isolated, cultured, and *ex vivo* challenged with antibiotic-sensitive (PaS) or multidrug-resistant (PaR) *Pseudomonas aeruginosa* strains or lipopolysaccharide (LPS). One day after the challenges, the levels of TNF-α, IL-1β, IL-6, CCL2, CXCL2, and CXCL10 were measured using ELISA. Data are presented as mean ± SEM. Statistical analysis was performed using Student’s t-test. The same control cohort was used for the experiments in **Figures 2**, **6**.

**Figure 3:**
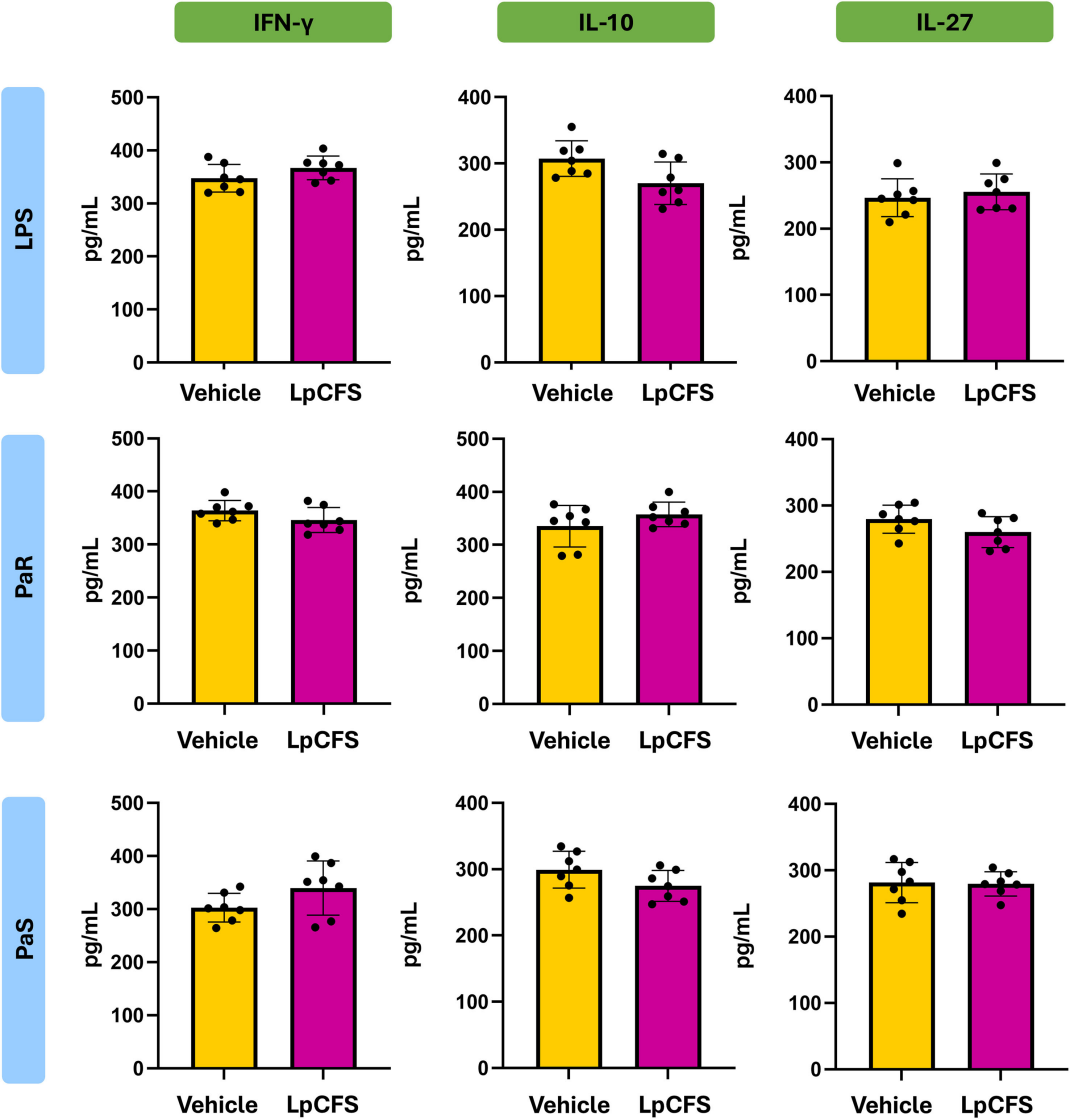
Effect of the administration of *Lactiplantibacillus plantarum* ATCC 10241 cell-free supernatant (LpCFS) on broncho-alveolar fluid (BAL) macrophage-enriched adherent cells. BALB/c mice (6-week-old) received aerosol administration of LpCFS or vehicle control for three consecutive days. On day four, BAL macrophage-enriched adherent cells were isolated, cultured, and *ex vivo* challenged with antibiotic-sensitive (PaS) or multidrug-resistant (PaR) *Pseudomonas aeruginosa* strains or lipopolysaccharide (LPS). One day after the challenges, the levels of IFN-γ, IL-10, and IL-27 were measured using ELISA. Data are presented as mean ± SEM. Statistical analysis was performed using Student’s t-test. Differences were considered statistically significant at *p* < 0.05 (*). The same control cohort was used for the experiments in **Figures 3**, **7**.

To determine whether LpCFS directly modulates innate immune responses, mice were treated by aerosol inhalation with LpCFS for three consecutive days. BAL macrophage-enriched adherent cells were subsequently isolated and cultured without any inflammatory stimuli. The production of the TNF-α, IL-1β, IL-6, IFN-γ, and the regulatory cytokines IL-10, and IL-27 were measured after 24 h (**Supplementary Figure 1**). No significant differences were found between the LpCFS and vehicle control groups for any of the studied cytokines. In addition, cultured BAL macrophage-enriched adherent cells were stimulated *ex vivo* with either PaS or PaR strains. In these experiments, lipopolysaccharide (LPS) challenge was used for comparisons. The production of the inflammatory cytokines and chemokines TNF-α, IL-1β, IL-6, CCL2, CXCL2, and CXCL10 (**Figure 2**), as well as IFN-γ, and the regulatory cytokines IL-10, and IL-27 (**Figure 3**) were measured after 24 h of the challenges.

Following stimulation with either LPS, PaS or PaR, no significant differences were observed in the production of inflammatory cytokines and chemokines, between BAL macrophage-enriched adherent cells isolated from LpCFS–treated mice and vehicle-treated animals (**Figure 2**). Similarly, LpCFS treatment did not significantly alter the levels of IFN-γ, or the regulatory–associated cytokines IL-10, or IL-27 under either stimulation condition (**Figure 3**).

These results indicate that LpCFS does not directly induce inflammatory activation, chemokine-driven immune cell recruitment, or regulatory cytokine responses in BAL macrophage-enriched adherent cells. This absence of direct immunomodulatory effects suggests that the protective activity of LpCFS observed previously *in vivo* (10) is unlikely to result from BAL macrophage-enriched adherent cells immune priming, but rather from mechanisms acting at the level of the pathogen during infection.

### Therapeutic aerosol administration of LpCFS reduces bacterial burden and attenuates lung injury following *P. aeruginosa* infection

**Figure 4:**
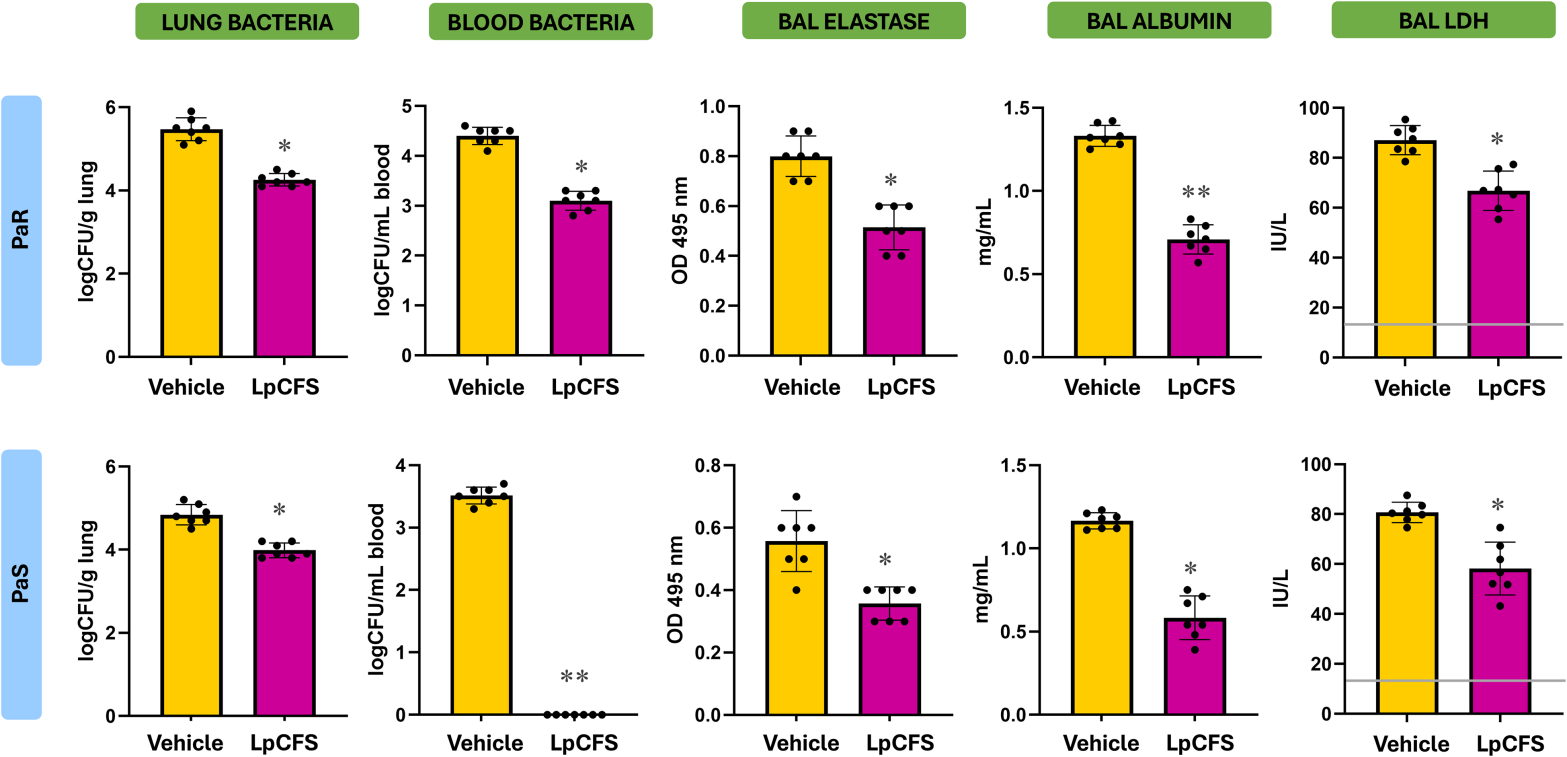
Effect of the therapeutic administration of *Lactiplantibacillus plantarum* ATCC 10241 cell-free supernatant (LpCFS) on the resistance to *Pseudomonas aeruginosa* respiratory infection. BALB/c mice (6-week-old) were challenged with antibiotic-sensitive (PaS) or multidrug-resistant (PaR) *P. aeruginosa* strains and then received aerosol administration of LpCFS or vehicle control for three consecutive days. Three days after the last LpCFS administration the lung and blood bacterial cell counts, and the levels of elastase, albumin, and lactate dehydrogenase (LDH) in broncho-alveolar fluid (BAL) samples were determined. Basal levels of LDH in BAL from uninfected mice receiving vehicle alone are included for comparison (gray line). BAL albumin and elastase levels are under the limit of detection in uninfected animals. Data are presented as mean ± SEM. Statistical analysis was performed using Student’s t-test. Differences were considered statistically significant at *p* < 0.05 (*), *p* < 0.05 (**).

We next evaluated the therapeutic potential of LpCFS *in vivo* using the murine models of *P. aeruginosa* lung infection. Mice were intranasally infected with either PaS or PaR strains and subsequently treated with aerosolized LpCFS for three consecutive days. Bacterial loads in lung and peripheral blood were quantified to assess pulmonary infection and systemic dissemination (**Figure 4**). LpCFS treatment significantly reduced bacterial counts in the lungs of mice infected with both PaS and PaR strains. Consistently, bacterial dissemination into the bloodstream was markedly decreased in LpCFS–treated mice compared with vehicle controls. Notably, LpCFS treatment completely prevented the development of bacteremia in PaS-infected mice (**Figure 4**).

To assess lung injury, levels of elastase, albumin, and lactate dehydrogenase (LDH) were measured in BAL samples of infected mice. Both PaS and PaR were capable of increasing the levels of BAL LDH compared to basal levels. In addition, elastase and albumin were detected in the BAL of infected mice in contrast to uninfected mice in which both parameters are under the detection limits. LpCFS–treated mice exhibited significantly lower levels of all three injury markers compared with vehicle-treated mice, indicating a more preserved alveolar capillary barrier integrity and reduced tissue damage following infection with both PaS and PaR (**Figure 4**).

**Figure 5:**
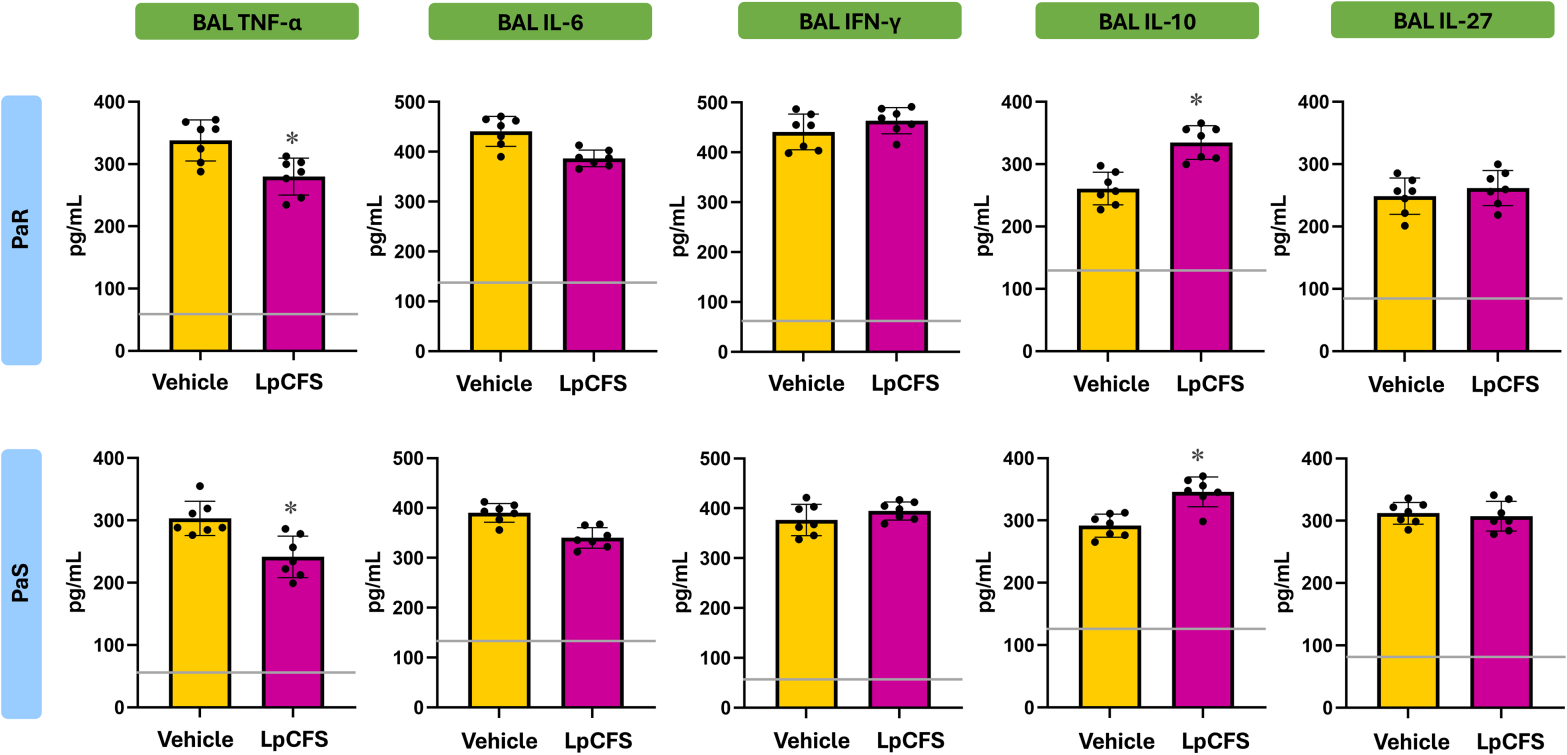
Effect of the therapeutic administration of *Lactiplantibacillus plantarum* ATCC 10241 cell-free supernatant (LpCFS) on the resistance to *Pseudomonas aeruginosa* respiratory infection. BALB/c mice (6-week-old) were challenged with antibiotic-sensitive (PaS) or multidrug-resistant (PaR) *P. aeruginosa* strains and then received aerosol administration of LpCFS or vehicle control for three consecutive days. Three days after the last LpCFS administration the levels of TNF-α, IL-6, IFN-γ, IL-10 and IL-27 in broncho-alveolar fluid (BAL) samples were determined. Basal levels of cytokines in BAL from uninfected mice receiving vehicle alone are included for comparison (gray lines). Data are presented as mean ± SEM. Statistical analysis was performed using Student’s t-test. Differences were considered statistically significant at *p* < 0.05 (*).

The analysis of BAL cytokines further demonstrated that LpCFS treatment induced a moderate change in cytokine profile. In mice infected with PaR, TNF-α was significantly reduced in LpCFS–treated mice while the regulatory cytokine IL-10 was significantly increased (**Figure 5**). A similar effect was observed in mice infected with PaS. In both models of *P. aeruginosa* respiratory infections, the LpCFS treatment was not able to modify the levels of IFN-γ or IL-27. These results indicate that LpCFS treatment attenuates lung damage caused by *P. aeruginosa* infection but modestly modify the respiratory cytokine profile.

### HK1505 modulates BAL macrophage-enriched adherent cells in a manner similar to live *L. rhamnosus* CRL1505

The ability of *L. rhamnosus* CRL1505 and HK1505 to beneficially modulate the immune response to respiratory viruses and Gram-positive bacterial pathogens was previously reported (5–7). However, their potential beneficial effects against Gram-negative bacterial pathogens have not been evaluated in depth. Then, in a second set of experiments, mice were intranasally treated for three consecutive days with either live *L. rhamnosus* CRL1505 (LV1505) or HK1505. BAL macrophage-enriched adherent cells were subsequently isolated and cultured without any inflammatory stimuli. The concentrations of TNF-α, IL-1β, IL-6, IFN-γ, IL-10, and IL-27 were measured after 24 h (**Supplementary Figure 2**). It was observed that BAL macrophage-enriched adherent cells obtained from both LV1505 and HK1505 groups produced significantly higher levels of all the cytokines evaluated compared to the cells obtained from vehicle control mice, which is in line with the immunomodulatory capacity of *L. rhamnosus* CRL1505.

**Figure 6:**
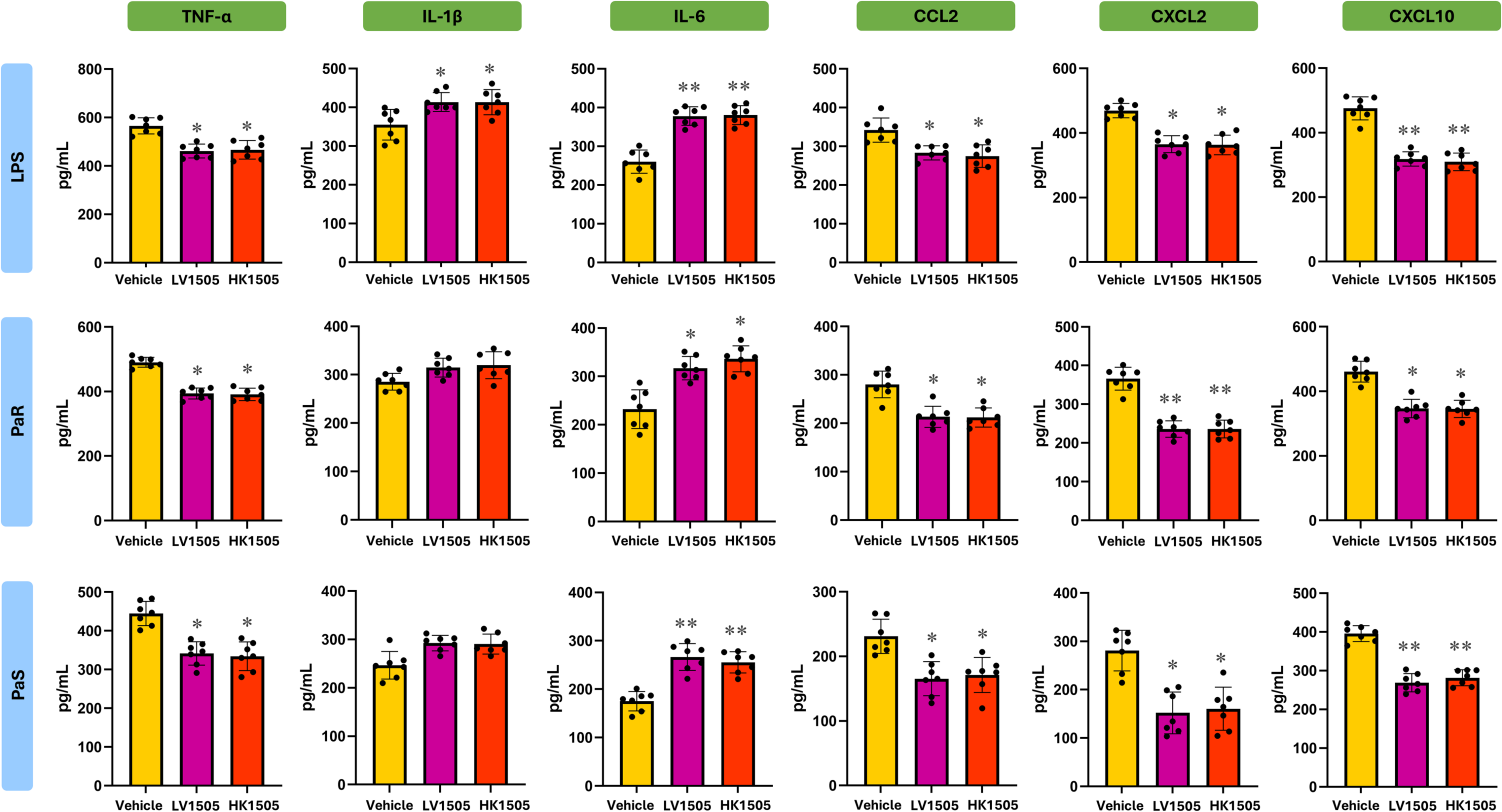
Effect of the administration of live (LV1505) and heat-killed (HK1505) *Lacticaseibacillus rhamnosus* CRL1505 on broncho-alveolar fluid (BAL) macrophage-enriched adherent cells. BALB/c mice (6-week-old) were nasally treated with LV1505, HK1505, or vehicle control for three consecutive days. On day four, BAL macrophage-enriched adherent cells were isolated, cultured, and *ex vivo* challenged with antibiotic-sensitive (PaS) or multidrug-resistant (PaR) *Pseudomonas aeruginosa* strains or lipopolysaccharide (LPS). One day after the challenges, the levels of TNF-α, IL-1β, IL-6, CCL2, CXCL2, and CXCL10 were measured using ELISA. Data are presented as mean ± SEM. Statistical analysis was performed using one-way analysis of variance (ANOVA), followed by Tukey’s multiple-comparison test. Differences were considered statistically significant at *p* < 0.05 (*), *p* < 0.01 (**). The same control cohort was used for the experiments in **Figures 2**, **6**.

**Figure 7:**
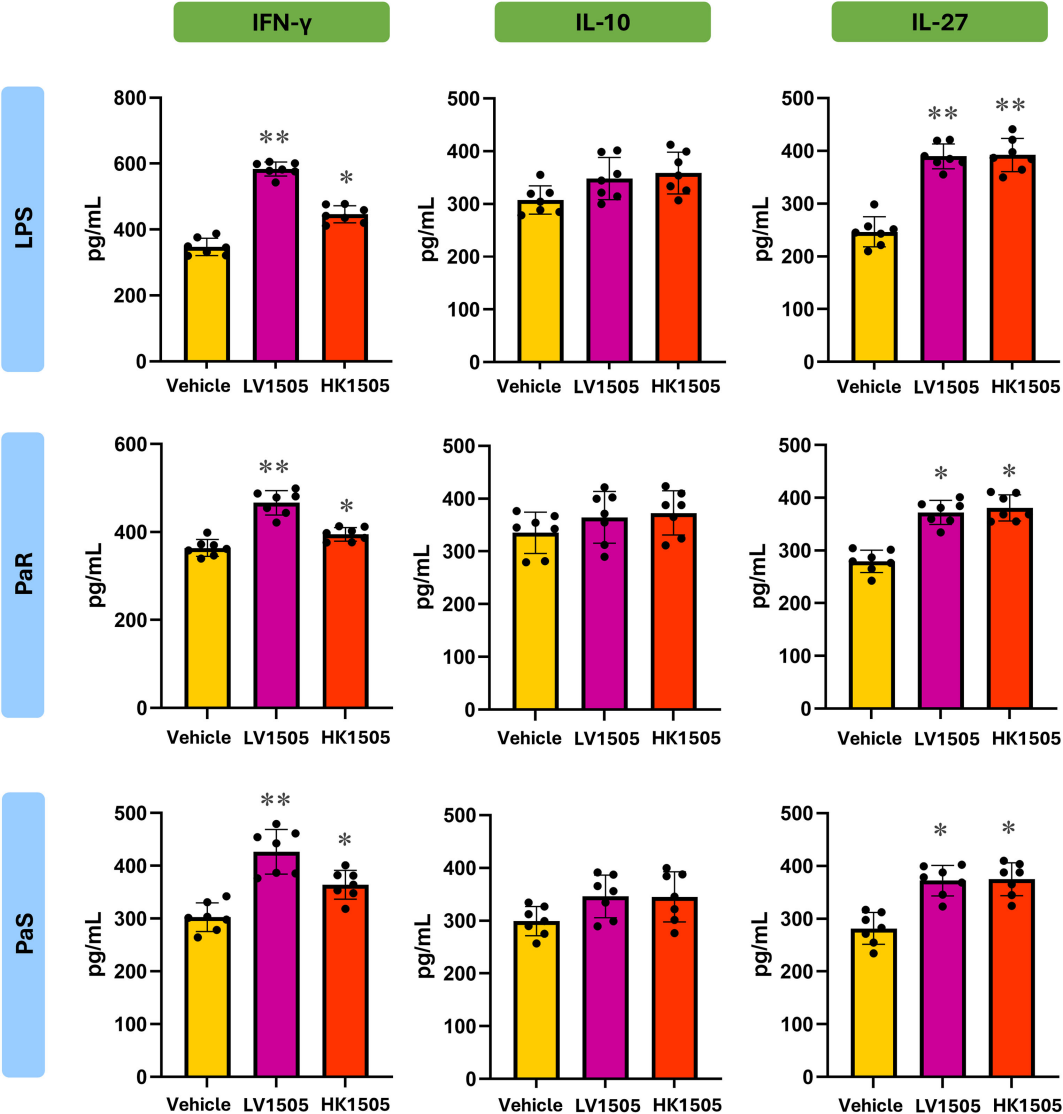
Effect of the administration of live (LV1505) and heat-killed (HK1505) *Lacticaseibacillus rhamnosus* CRL1505 on broncho-alveolar fluid (BAL) macrophage-enriched adherent cells. BALB/c mice (6-week-old) were nasally treated with LV1505, HK1505, or vehicle control for three consecutive days. On day four, BAL macrophage-enriched adherent cells were isolated, cultured, and *ex vivo* challenged with antibiotic-sensitive (PaS) or multidrug-resistant (PaR) *Pseudomonas aeruginosa* strains or lipopolysaccharide (LPS). One day after the challenges, the levels IFN-γ, IL-10, and IL-27 were measured using ELISA. Data are presented as mean ± SEM. Statistical analysis was performed using one-way analysis of variance (ANOVA), followed by Tukey’s multiple-comparison test. Differences were considered statistically significant at *p* < 0.05 (*), *p* < 0.01 (**). The same control cohort was used for the experiments in **Figures 3**, **7**.

In addition, BAL macrophage-enriched adherent cells were stimulated *ex vivo* with LPS, PaS or PaR and cytokines were evaluated in culture supernatants 24 h after the challenges (**Figures 6**, **7**). Both LV1505 and HK1505 treatments were able to induce a reduction in the production of TNF-α, CCL2, CXCL2, and CXCL10 and an increase in IL-6 in LPS-, PaS- and PaR-challenged BAL macrophage-enriched adherent cells (**Figure 6**). In addition, LV1505 and HK1505 increased the capacity of cells to produce IL-1β in response to LPS stimulation. In parallel, BAL macrophage-enriched adherent cells isolated from both LV1505- and HK1505-treated mice exhibited a marked capacity to produce IFN-γ and IL-27 in response to LPS, PaS and PaR challenges, compared with vehicle-treated mice (**Figure 7**). Neither LV1505 nor HK1505 induced changes in IL-10 levels.

Importantly, no significant differences were observed between BAL macrophage-enriched adherent cells isolated from LV1505- and HK1505-treated mice across either cytokine module, indicating that heat inactivation does not impair the immunostimulatory capacity of *L. rhamnosus* CRL1505. These results demonstrate that HK1505 would prime BAL macrophage-enriched adherent cells toward an enhanced immunomodulatory cytokine profile that would mediate an enhanced resistance to *P. aeruginosa* infection.

### Prophylactic administration of HK1505 improves outcomes of *P. aeruginosa* lung infection

**Figure 8:**
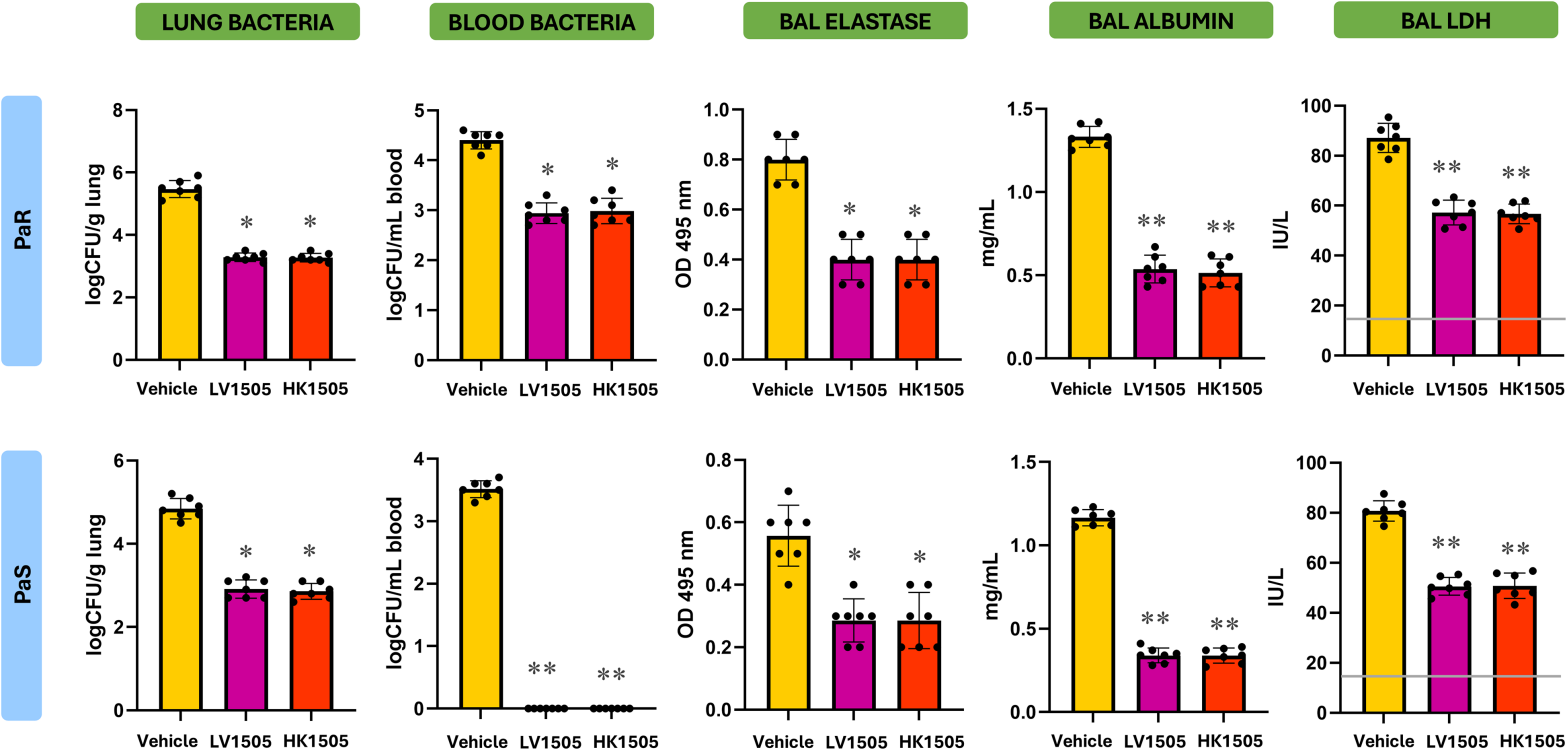
Effect of the prophylactic administration live (LV1505) and heat-killed (HK1505) *Lacticaseibacillus rhamnosus* CRL1505 on the resistance to *Pseudomonas aeruginosa* respiratory infection. BALB/c mice (6-week-old) received prophylactic intranasal administration of LV1505 or HK1505 for three days and then were challenged with antibiotic-sensitive (PaS) or multidrug-resistant (PaR) *P. aeruginosa* strains. Three days after the infection, the lung and blood bacterial cell counts, and the levels of elastase, albumin, and lactate dehydrogenase (LDH) in broncho-alveolar fluid (BAL) samples were determined. Basal levels of LDH in BAL from uninfected mice receiving vehicle alone are included for comparison (gray line). BAL albumin and elastase levels are under the limit of detection in uninfected animals. Data are presented as mean ± SEM. Statistical analysis was performed using one-way analysis of variance (ANOVA), followed by Tukey’s multiple-comparison test. Differences were considered statistically significant at *p* < 0.05 (*), *p* < 0.05 (**).

Considering the *ex vivo* results explained in the previous paragraph, we next examined whether the immune priming effects of LV1505 and HK1505 translated into improved resistance to *P. aeruginosa* infection *in vivo*. Then, mice were prophylactically treated with LV1505 or HK1505 prior to the intranasal infection with PaS or PaR strains. Both LV1505 and HK1505 significantly reduced bacterial loads in lung and peripheral blood following infection (**Figure 8**). Remarkably, LV1505- and HK1505-treated mice had negative hemocultures after the infection with PaS strain. In line with the lower bacterial loads, the three markers of lung damage, BAL elastase, albumin, and LDH, were markedly lower in LV1505- or HK1505-treated mice compared with vehicle-treated animals (**Figure 8**).

**Figure 9:**
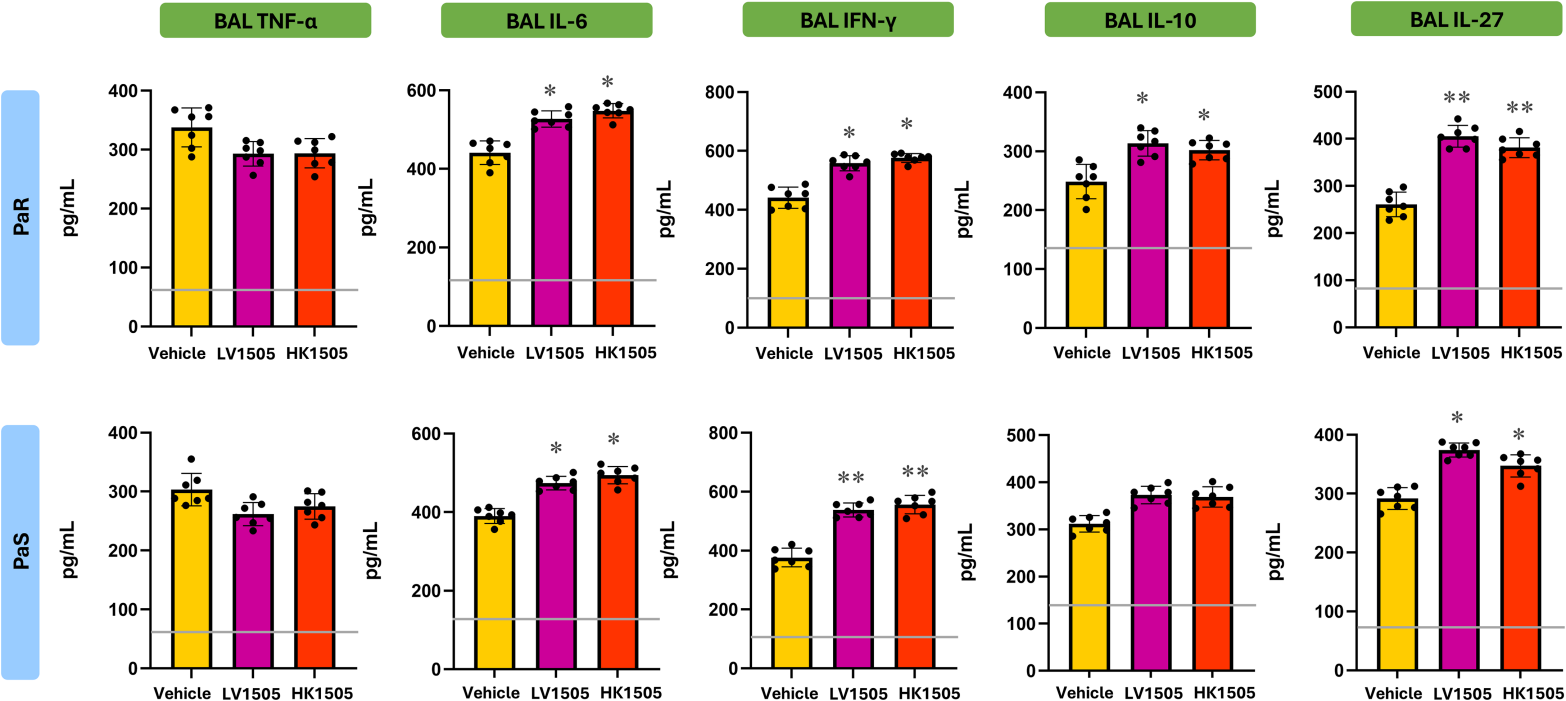
Effect of the prophylactic administration live (LV1505) and heat-killed (HK1505) *Lacticaseibacillus rhamnosus* CRL1505 on the resistance to *Pseudomonas aeruginosa* respiratory infection. BALB/c mice (6-week-old) received prophylactic intranasal administration of LV1505 or HK1505 for three days and then were challenged with antibiotic-sensitive (PaS) or multidrug-resistant (PaR) *P. aeruginosa* strains. Three days after the infection, the levels of TNF-α, IL-6, IFN-γ, IL-10 and IL-27 in broncho-alveolar fluid (BAL) samples were determined. Basal levels of cytokines in BAL from uninfected mice receiving vehicle alone are included for comparison (gray lines). Data are presented as mean ± SEM. Statistical analysis was performed using one-way analysis of variance (ANOVA), followed by Tukey’s multiple-comparison test. Differences were considered statistically significant at *p* < 0.05 (*), *p* < 0.05 (**).

The analysis of BAL cytokines demonstrated a clear immunomodulatory effect of LV1505 or HK1505 (**Figure 9**). It was observed that mice treated with either LV1505 or HK1505 had significantly higher levels of respiratory IFN-γ, IL-6, and IL-27 compared with vehicle-treated mice, in both PaS- and PaR-infected groups. In addition, the levels of BAL IL-10 were higher in LV1505- or HK1505-treated mice compared with vehicle-treated animals after the infection with PaR (**Figure 9**). Then, the results show that LV1505 and HK1505 are capable of improving the resistance to *P. aeruginosa* infection by enhancing the respiratory immune response and importantly, the effectiveness of HV1505 is comparable to the observed for live *L. rhamnosus* CRL1505.

### Combined prophylactic and therapeutic treatments provide superior protection against *P. aeruginosa* infection

**Figure 10:**
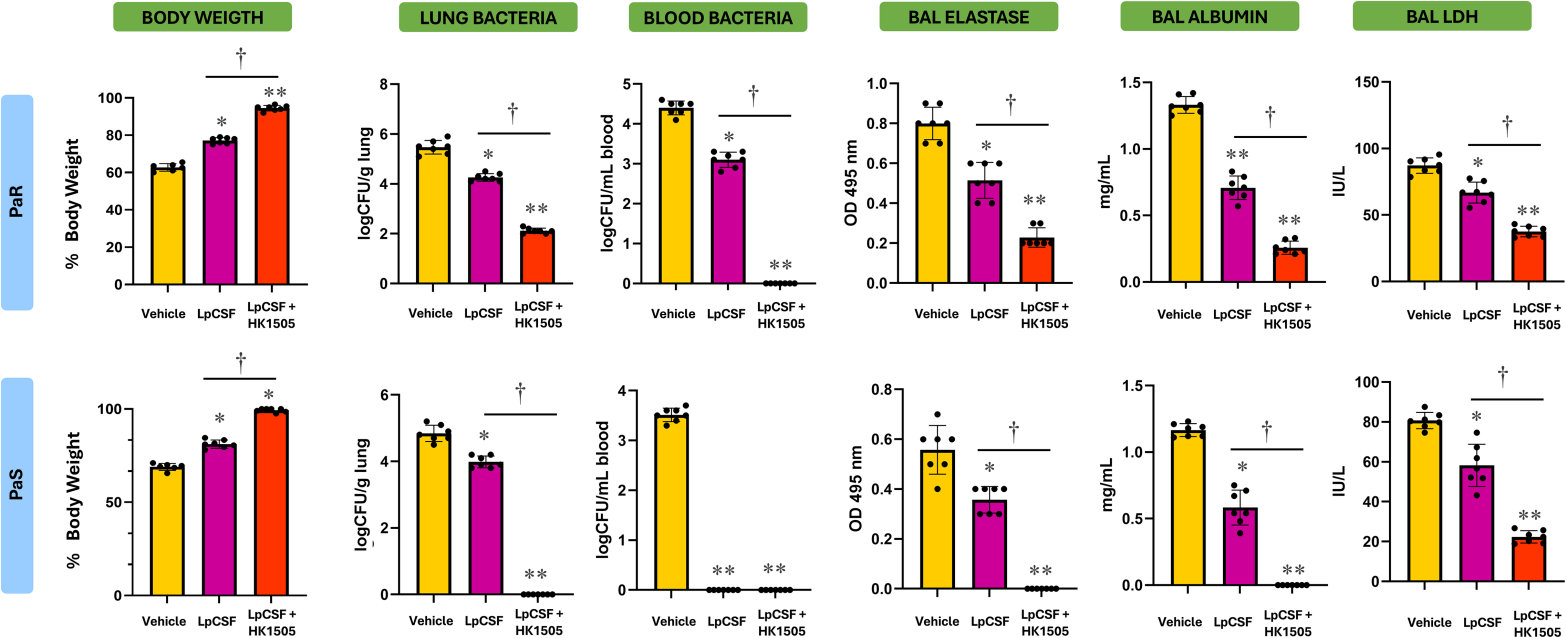
Effect of the combined prophylactic administration of heat-killed (HK1505) *Lacticaseibacillus rhamnosus* CRL1505 and the therapeutic administration of *Lactiplantibacillus plantarum* ATCC 10241 cell-free supernatant (LpCFS) on the resistance to *Pseudomonas aeruginosa* respiratory infection. BALB/c mice (6-week-old) received prophylactic intranasal administration of HK1505 for three days, then were challenged with antibiotic-sensitive (PaS) or multidrug-resistant (PaR) *P. aeruginosa* strains and received aerosol administration of LpCFS or vehicle control for three consecutive days after the infection. Three days after the last LpCFS administration, body weight change, the lung and blood bacterial cell counts, and the levels of elastase, albumin, and lactate dehydrogenase (LDH) in broncho-alveolar fluid (BAL) samples were determined.). Data are presented as mean ± SEM. Statistical analysis was performed using Student’s t-test. Differences were considered statistically significant at *p* < 0.05 (*), *p* < 0.05 (**) compared with vehicle control. † significant at *p* < 0.05 (*) between the indicated groups.

Finally, we evaluated the protective efficacy of combining prophylactic HK1505 treatment with therapeutic aerosol administration of LpCFS. In this set of experiments, we used as controls a group of mice receiving only the therapeutic LpCFS treatment. It was shown that animals receiving the combined HK1505-LpCFS treatment exhibited the most pronounced protective effects against both PaS and PaR infections (**Figure 10**). Body weight evolution was also monitored as an indicator of disease severity. Mice receiving the combined HK1505-LpCFS treatment exhibited reduced weight loss compared with LpCFS and vehicle control animals following PaS or PaR infections (**Figure 10**). In PaS-infected mice, bacterial counts in lung and blood were below the detection limit following combined treatment, indicating near-complete bacterial clearance. In PaR-infected mice, the combined HK1505-LpCFS treatment effectively prevented the development of bacteremia and significantly reduced pulmonary bacterial burden compared with LpCFS treatment alone. In addition, the markers of lung injury were significantly reduced in mice receiving the combined HK1505-LpCFS treatment, indicating enhanced protection of the lung tissue compared with LpCFS alone (**Figure 10**). Remarkably, the levels of BAL elastase and albumin were below the respective detection limits in mice receiving the HK1505-LpCFS treatment and infected with PaS.

**Figure 11:**
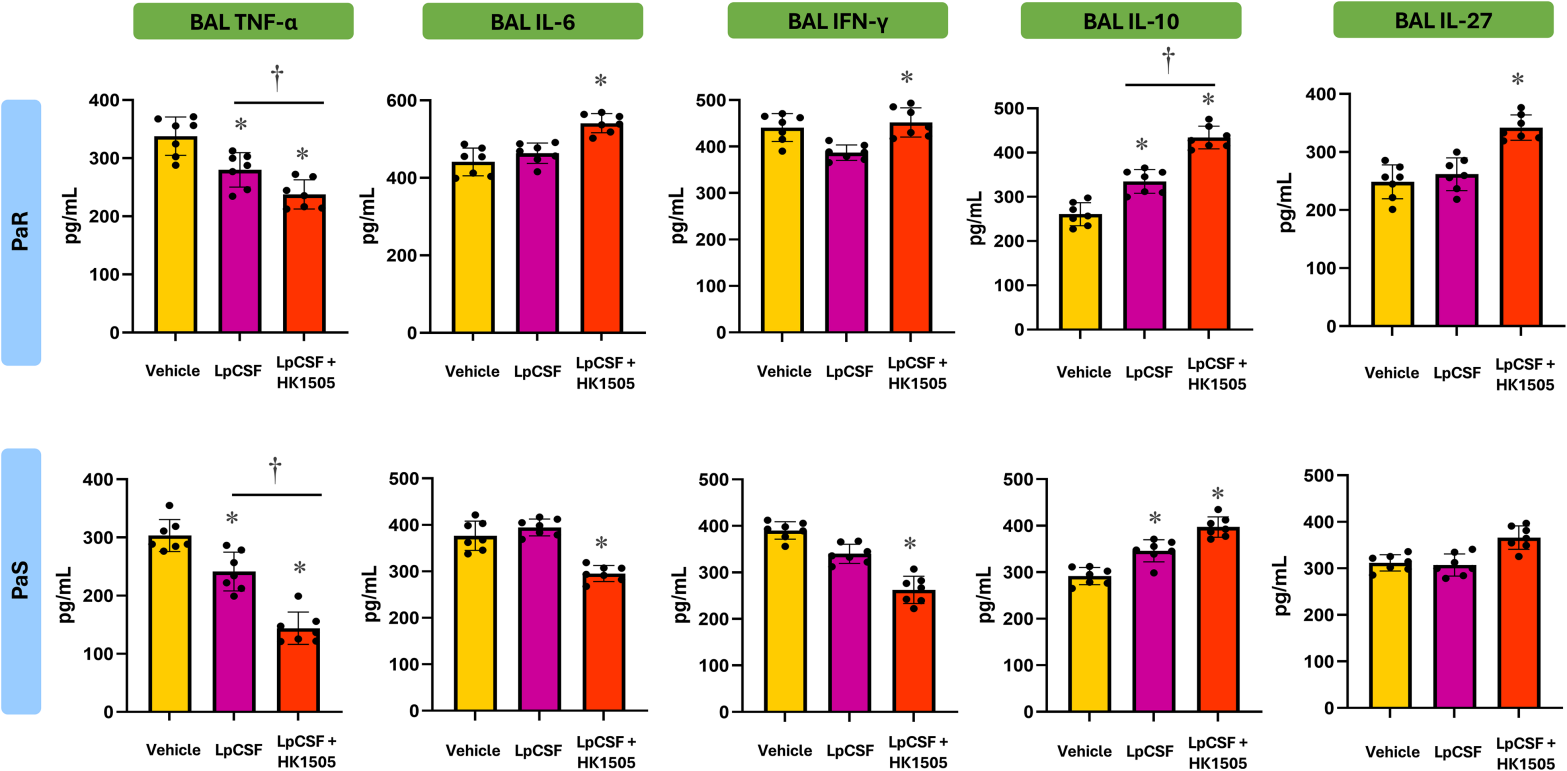
Effect of the combined prophylactic administration of heat-killed (HK1505) *Lacticaseibacillus rhamnosus* CRL1505 and the therapeutic administration of *Lactiplantibacillus plantarum* ATCC 10241 cell-free supernatant (LpCFS) on the resistance to *Pseudomonas aeruginosa* respiratory infection. BALB/c mice (6-week-old) received prophylactic intranasal administration of HK1505 for three days, then were challenged with antibiotic-sensitive (PaS) or multidrug-resistant (PaR) *P. aeruginosa* strains and received aerosol administration of LpCFS or vehicle control for three consecutive days after the infection. Three days after the last LpCFS administration, the levels of TNF-α, IL-6, IFN-γ, IL-10 and IL-27 in broncho-alveolar fluid (BAL) samples were determined. Data are presented as mean ± SEM. Statistical analysis was performed using Student’s t-test. Differences were considered statistically significant at *p* < 0.05 (*), *p* < 0.05 (**) compared with vehicle control. † significant at *p* < 0.05 (*) between the indicated groups.

When the respiratory cytokines were analyzed, a distinct effect of the combined HK1505-LpCFS treatment was revealed for each infection model (**Figure 11**). In PaR-infected mice, the combined HK1505-LpCFS administration enhanced IFN-γ and IL-6 together with improvements of the regulatory cytokines IL-10 and IL-27 compared with LpCFS alone. On the other hand, PaS-infected mice receiving the combined treatment exhibited reduced levels of TNF-α, IL-6, as well as IFN-γ levels in the respiratory tract compared to animals in the LpCFS alone group. In these mice, the HK1505-LpCFS treatment was not able to induce changes in the levels of IL-10 or IL-27 compared with the controls (**Figure 11**).

## Discussion

The results obtained in this study allow us to draw three relevant conclusions. *a)* Our previous studies demonstrated that nasal administration of LV1505 or HK1505 enhances respiratory defenses against viral and Gram-positive bacterial pathogens (5, 6). The present study further shows protective effects against a Gram-negative respiratory pathogen. These findings suggest that nasal priming with immunobiotics such as *L. rhamnosus* CRL1505 or its derived postbiotic HK1505 modulates respiratory innate immune mechanisms shared across pathogens, thereby improving protection against a broad range of microbes. Notably, LV1505 and HK1505 exhibited comparable protective effects. Therefore, particular attention should be given to the heat-killed preparation, as non-viable microbial products may represent a safer strategy for immunocompromised individuals—who are frequently infected with *P. aeruginosa*—where the use of live microorganisms requires caution. *b)* We previously reported that LpCFS inhibits biofilm formation and virulence in *P. aeruginosa* (9, 10), and that nebulization with LpCFS reduces lung inflammatory damage in infected mice (10). Here, we extend those findings by showing that, despite its protective effects *in vivo*, LpCFS does not directly modulate the respiratory immune response *ex vivo*. These findings suggest that LpCFS primarily exerts pathogen-directed effects rather than acting as a direct immunostimulatory agent. Notably, although HK1505 and LpCFS produced broadly comparable antibacterial outcomes, their cytokine and chemokine profiles differed. While LpCFS likely influences inflammatory mediators indirectly through reduced bacterial burden, HK1505 directly modulates host immune cells. Therefore, while both approaches ultimately result in improved control of infection, they may operate through partially different mechanisms. The third conclusion is related to the demonstration that: *c)* a dual non-viable microbial strategy integrating a prophylactic immune priming with HK1505 and a therapeutic administration of LpCFS provides effective protection against both antibiotic-sensitive and multidrug-resistant *P. aeruginosa* lung infection. By simultaneously enhancing pulmonary innate immune readiness and directly targeting bacterial virulence, this approach significantly reduces bacterial burden, limits lung injury, and maintains controlled inflammatory responses. Importantly, all protective effects were achieved without the use of live bacteria, highlighting the translational potential of this safe microbial preventive/therapeutic strategy for managing antibiotic-resistant respiratory infections in immunocompromised patients in which the use of live microorganism could be deleterious.

*Ex vivo* experiments were performed using primary cultures of BAL macrophage-enriched adherent cells. Although this population is widely considered macrophage-dominant, the presence of other recruited myeloid cells after probiotic/postbiotic treatments cannot be completely excluded. However, previous studies with these probiotic/postbiotic preparations did not detect airway inflammation or leukocyte recruitment (6), suggesting minimal changes in cellular composition. Thus, while this limitation should be considered, our working hypothesis is that alveolar macrophages are likely the principal cells modulated by LV1505 and HK1505.

Alveolar macrophages are central regulators of pulmonary innate immunity, coordinating pathogen sensing, antimicrobial defense, and inflammatory homeostasis (11–17). They promote rapid bacterial clearance through phagocytosis and killing, while regulating chemokine production to limit excessive inflammatory cell recruitment (18–22). Macrophage sensing through pattern-recognition receptors such as TLR4 is also critical for controlling *P. aeruginosa* infection while preventing immunopathology (23–25). Conversely, dysregulated macrophage activation can aggravate lung injury, particularly during infections with biofilm-forming and antibiotic-resistant strains (26). In light of these studies, the selective immune priming induced by HK1505, characterized by enhanced production of antimicrobial and regulatory/effector cytokines coupled with restrained chemokine expression, aligns with an immune profile optimized for alveolar macrophage–mediated protection (5, 12). Rather than broadly amplifying inflammation, HK1505 appears to reinforce macrophage-intrinsic defense mechanisms that are effective across viral, Gram-positive, and Gram-negative bacterial respiratory infections. This macrophage-centered mode of action provides a mechanistic framework for the broad protective effects of HK1505 observed in previous works (6) and in the present study, supporting its potential utility as a prophylactic immunomodulator against diverse pulmonary pathogens. Consistent with these findings, several studies have shown that nasal postbiotic administration enhances respiratory host defenses through modulation of alveolar macrophages (27–29). Postbiotics derived from *Lactobacillus gasseri* or *Ligilactobacillus salivarius* strains improve resistance to viral and bacterial respiratory infections by modulating TLR-mediated responses and increasing IFN-β, IFN-γ, IL-10, and IL-27 levels in the respiratory tract (27, 28).

HK1505 protected mice primarily through modulation of innate immunity. Prophylactic administration primed respiratory immune cells to enhance antimicrobial cytokine production upon bacterial challenge. Concurrently, HK1505 reduced chemokine expression (CCL2, CXCL2, CXCL10), limiting monocyte and neutrophil recruitment. This cytokine–chemokine profile suggests that HK1505 promotes a localized, cell-intrinsic antimicrobial response while limiting excessive immune cell infiltration, a balance that is critical for effective pathogen clearance without inducing immunopathology. The dissociation between elevated TNF-α levels and reduced lung injury in HK1505-treated mice is notable. Although TNF-α is often linked to pulmonary damage, its effects are context dependent (30). When coupled with controlled chemokine production and regulatory mediators such as IL-27, it can enhance antimicrobial defense without excessive inflammation (30–32). Together, these findings indicate that HK1505 induces a qualitatively distinct immune state characterized by enhanced antimicrobial capacity and restrained inflammatory amplification. Differences observed between antibiotic-sensitive and multidrug-resistant *P. aeruginosa* infections further support this interpretation. In PaR-infected mice, HK1505 treatment resulted in increased IL-10 production, whereas this effect was not observed in PaS infection. This selective induction of IL-10 may reflect prolonged or heightened inflammatory pressure associated with PaR infection due to its enhanced biofilm-forming capacity and resistance to clearance (10). Since IL-10 is a critical anti-inflammatory cytokine that limits tissue damage during sustained immune activation (33), the absence of elevated IL-10 in PaS-infected mice may indicate that bacterial clearance occurred more rapidly, allowing inflammation to resolve before sampling. Thus, HK1505 appears capable of dynamically modulating regulatory pathways in response to infection severity.

**Figure 12:**
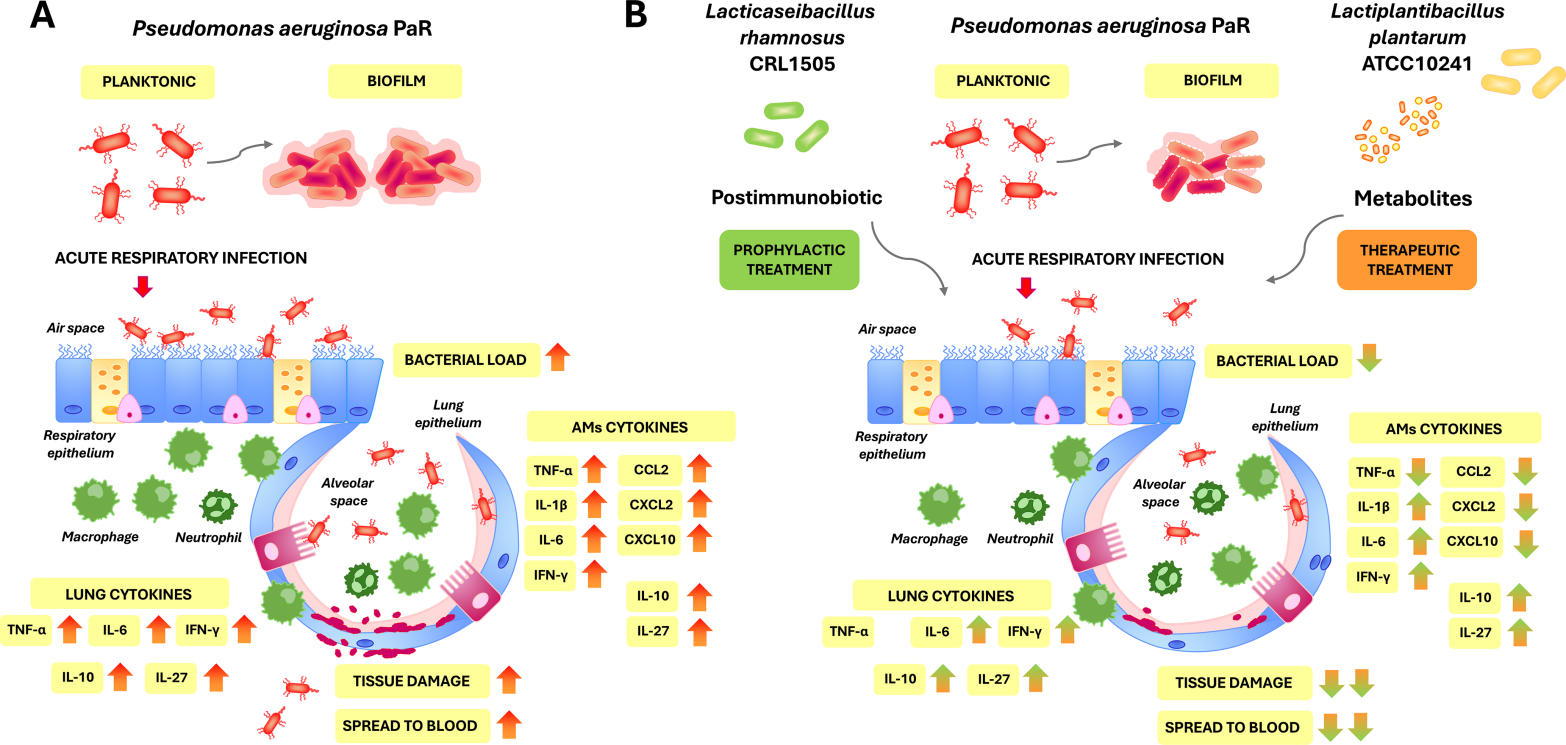
Hypothetical model illustrating possible mechanisms of the dual host- and pathogen-targeted probiotic strategy against *Pseudomonas aeruginosa* respiratory infection. **(A)** Effect of multidrug-resistant (PaR) *P. aeruginosa* infection on lung injury and innate immune response. **(B)** This schematic summarizes the proposed model underlying the protective effects of the combined prophylactic administration of heat-killed (HK1505) *Lacticaseibacillus rhamnosus* CRL1505 and the therapeutic administration of *Lactiplantibacillus plantarum* ATCC 10241 cell-free supernatant (LpCFS) on the resistance to *P. aeruginosa* respiratory infection. The combined use of HK1505 and LpCFS integrates host-directed immunomodulation with pathogen-directed anti-virulence activity, resulting in enhanced bacterial clearance, reduced lung epithelial injury, controlled inflammatory responses, and improved infection outcomes. This schematic representation summarizes the potential mechanisms suggested by our findings and by previous studies. The pathways illustrated should be considered as a working model rather than experimentally demonstrated mechanisms.

On the other hand, we demonstrated previously that LpCFS has direct anti-virulence activity against *P. aeruginosa* (9, 10). LpCFS was shown to attenuate pathogenicity in both PaS and PaR strains by inhibiting biofilm formation, suppressing quorum sensing–regulated virulence factor production, and reducing bacterial viability. These pathogen-directed effects are particularly relevant for PaR strain, in which enhanced biofilm formation contributes to antibiotic resistance, prolonged colonization, and increased tissue damage (**Figure 12A**) (10). In this work, we showed that both PaS and PaR can trigger inflammation in the respiratory tract of mice although with differences between them. Distinct virulence-associated traits, including biofilm-forming capacity, may contribute to the distinct inflammatory responses observed for PaS and PaR. The results show that antibiotic-resistant and -susceptible *P. aeruginosa* strains may trigger distinct respiratory inflammatory responses, however, it should be noted that only one antibiotic-susceptible and one antibiotic-resistant *P. aeruginosa* strains were evaluated in the present study, which limits the generalization of these conclusions. In addition, we showed here that LpCFS effectively reduced bacterial loads and attenuated lung damage, likely through a combination of direct bactericidal activity and inhibition of biofilm formation. Biofilms represent a critical virulence mechanism of *P. aeruginosa*, particularly in multidrug-resistant strains, where they confer tolerance to antibiotics and shield bacteria from immune-mediated clearance (4, 34). By weakening biofilm integrity, LpCFS may render bacteria more susceptible to both host defense mechanisms and physical clearance, thereby improving infection outcomes without amplifying inflammatory responses.

By impairing bacterial persistence and virulence independently of host immunity, LpCFS complements the immunomodulatory effects induced by HK1505, providing a coordinated strategy that targets both host defense capacity and pathogen fitness (**Figure 12B**). This dual mode of action likely underlies the superior protection observed with combined treatment and highlights the therapeutic potential of non-viable microbial products as alternatives or adjuncts to conventional antibiotics. The combined use of HK1505 and LpCFS yielded the most pronounced protective effects, underscoring the complementary mechanisms of immune priming and pathogen-directed therapy. In PaS infection, combined treatment resulted in near-complete bacterial clearance and reduced inflammatory cytokine levels, consistent with progression toward resolution of infection. In PaR infection, combined treatment prevented bacteremia and significantly improved lung outcomes while maintaining elevated levels of IL-6, IFN-γ, IL-10, and IL-27, indicative of a robust yet tightly regulated immune response. These findings highlight the importance of tailoring immune responses to the pathogenic context, particularly in infections caused by multidrug-resistant, biofilm-forming bacteria.

From a translational perspective, the use of non-viable immunomodulatory agents represents a key advantage of this strategy (38, 39). HK1505 and LpCFS avoid concerns associated with live bacteria, including uncontrolled colonization, horizontal gene transfer, and safety risks in immunocompromised patients. Both can be standardized, stored, and delivered by aerosol, making them suitable for clinical settings such as intensive care units or patients at risk of ventilator-associated pneumonia. This approach also aligns with growing interest in postbiotics as safe modulators of host–microbe interactions. However, this study was conducted in immunocompetent hosts, and because HK1505 acts primarily through immune modulation, its efficacy may depend on host immune status. This is particularly relevant since *P. aeruginosa* infections commonly occur in immunocompromised individuals, including patients with HIV, chronic liver disease, or immune dysregulation such as cystic fibrosis (35–37). Under conditions of compromised immunity, the capacity of HK1505 to prime innate immune responses may be altered, potentially limiting its protective effects. In this context, the pathogen-directed activity of LpCFS may become particularly relevant, as it does not rely on host immune competence to impair bacterial growth and virulence. This distinction further supports the rationale for the combined host- and pathogen-targeted strategy proposed in this study. Future investigations should therefore evaluate the efficacy of HK1505 and LpCFS in models of immune impairment. Such studies would be essential to define the limits of immunobiotic-based interventions and to determine whether pathogen-targeted postbiotics alone or in combination with immune modulation can provide protection in vulnerable host populations.

In conclusion, our study provides proof-of-concept evidence that integrating prophylactic immune priming with therapeutic antibiofilm intervention offers an effective, non-antibiotic strategy for controlling antimicrobial-resistant *P. aeruginosa* lung infection. By enhancing pulmonary innate immune defenses and limiting immunopathology and directly targeting bacterial virulence, this dual postbiotic approach addresses key challenges associated with multidrug-resistant respiratory pathogens. These findings support further exploration of postbiotic-based immunomodulatory therapies as adjuncts or alternatives to conventional antibiotics in the management of severe lung infections. Because intranasal instillation and aerosol exposure differ in terms of dose delivery, anatomical deposition and compound stability, the results obtained with these two approaches should not be interpreted as directly comparable. Further studies will be necessary to optimize delivery strategies that allow a more precise and practical administration of these immunomodulatory and antimicrobial preparations for potential translational applications.

## Data Availability

The original contributions presented in the study are included in the article/**Supplementary Material**. Further inquiries can be directed to the corresponding authors.

## Author’s note

Leonardo Albarracin: Japan Society for the Promotion of Science (JSPS) Postdoctoral Fellow.

## Supplementary material

Supplementary Figure 1 Effect of the administration of *Lactiplantibacillus plantarum* ATCC 10241 cell-free supernatant (LpCFS) on broncho-alveolar fluid (BAL) macrophage-enriched adherent cells. BALB/c mice (6-week-old) received aerosol administration of LpCFS or vehicle control for three consecutive days. On day four, BAL macrophage-enriched adherent cells were isolated and cultured. One day after, the levels of TNF-α, IL-1β, IL-6, IFN-γ, IL-10 and IL-27 were measured using ELISA. Data are presented as mean ± SEM. Statistical analysis was performed using Student’s t-test. Differences were considered statistically significant at *p* < 0.05 (*). The same control cohort was used for the experiments in **Supplementary Figures 1**, **2**.

Supplementary Figure 2 Effect of the administration of live (LV1505) and heat-killed (HK1505) *Lacticaseibacillus rhamnosus* CRL1505 on broncho-alveolar fluid (BAL) macrophage-enriched adherent cells. BALB/c mice (6-week-old) were nasally treated with LV1505, HK1505, or vehicle control for three consecutive days. On day four, BAL macrophage-enriched adherent cells were isolated and cultured. One day after, the levels of TNF-α, IL-1β, IL-6, IFN-γ, IL-10 and IL-27 were measured using ELISA. Data are presented as mean ± SEM. Statistical analysis was performed using Student’s t-test. Differences were considered statistically significant at *p* < 0.05 (*), *p* < 0.05 (**). The same control cohort was used for the experiments in **Supplementary Figures 1**, **2**.
