# Supplementary figures and images for: Prophylactic immune priming with heat-killed Lacticaseibacillus rhamnosus combined with therapeutic Lactiplantibacillus plantarum cell-free supernatant protects against Pseudomonas aeruginosa lung infection in mice
Source: Front Immunol. 2026 Apr 1;17:1802599. doi: 10.3389/fimmu.2026.1802599 (PMC13079012; doi:10.3389/fimmu.2026.1802599)

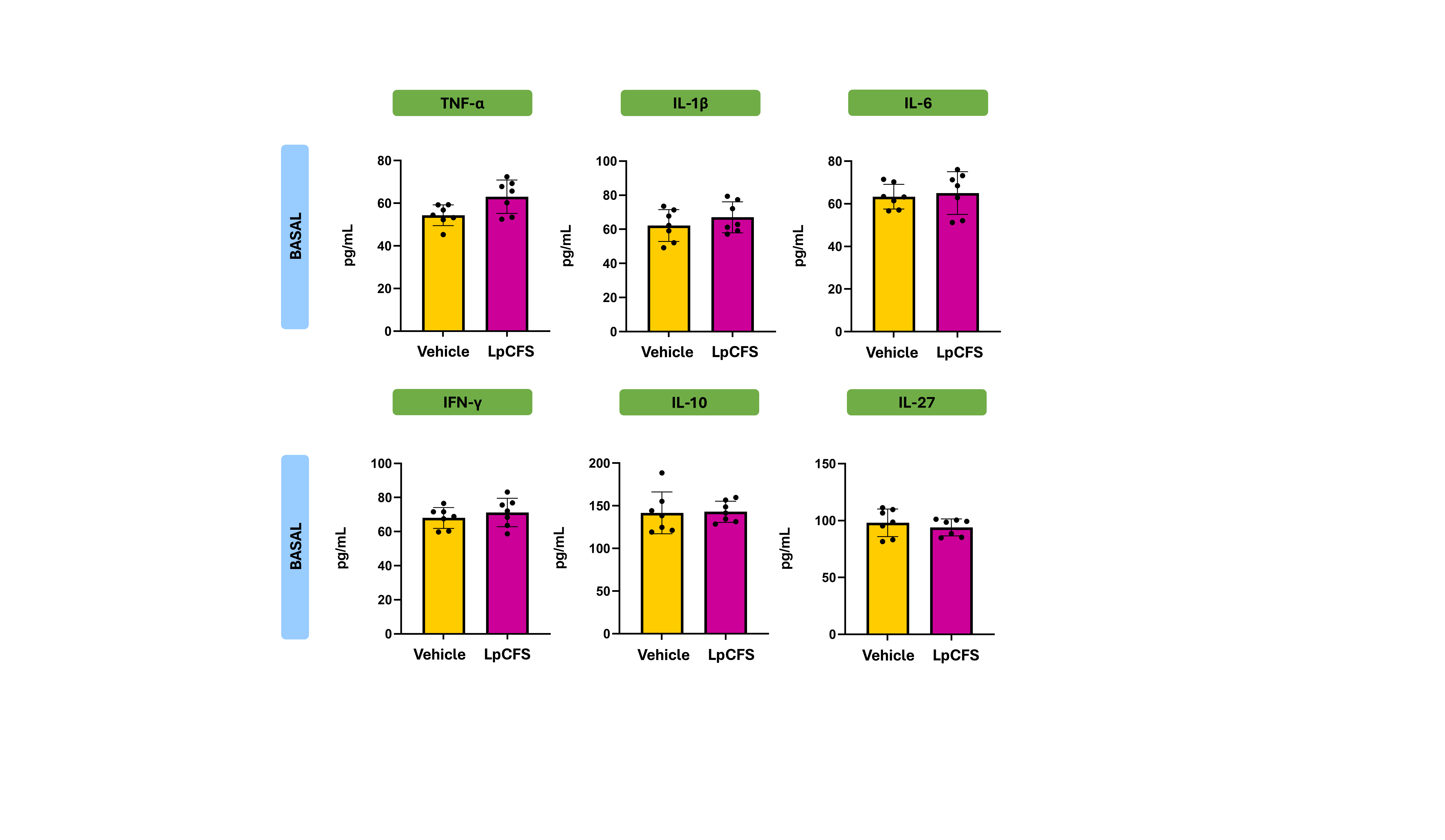

Supplement: Supplementary Figure 1 — Effect of the administration of Lactiplantibacillus plantarum ATCC 10241 cell-free supernatant (LpCFS) on broncho-alveolar fluid (BAL) macrophage-enriched adherent cells. BALB/c mice (6-week-old) received aerosol administration of LpCFS or vehicle control for three consecutive days. On day four, BAL macrophage-enriched adherent cells were isolated and cultured. One day after, the levels of TNF-α, IL-1β, IL-6, IFN-γ, IL-10 and IL-27 were measured using ELISA. Data are presented as mean ± SEM. Statistical analysis was performed using Student’s t-test. Differences were considered statistically significant at p < 0.05 (*). The same control cohort was used for the experiments in Supplementary Figures 1, 2. [file Image1.tif]

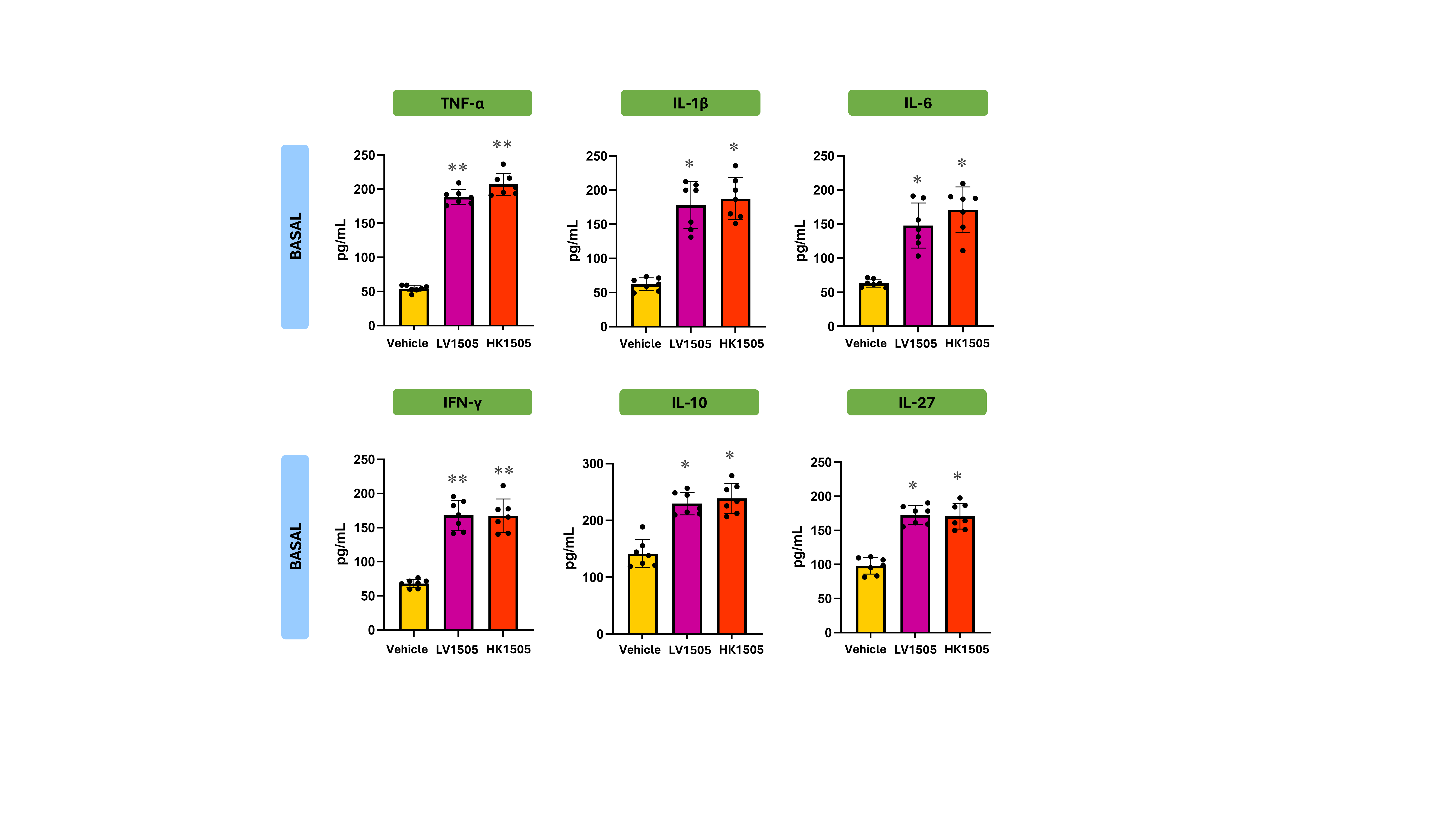

Supplement: Supplementary Figure 2 — Effect of the administration of live (LV1505) and heat-killed (HK1505) Lacticaseibacillus rhamnosus CRL1505 on broncho-alveolar fluid (BAL) macrophage-enriched adherent cells. BALB/c mice (6-week-old) were nasally treated with LV1505, HK1505, or vehicle control for three consecutive days. On day four, BAL macrophage-enriched adherent cells were isolated and cultured. One day after, the levels of TNF-α, IL-1β, IL-6, IFN-γ, IL-10 and IL-27 were measured using ELISA. Data are presented as mean ± SEM. Statistical analysis was performed using Student’s t-test. Differences were considered statistically significant at p < 0.05 (*), p < 0.05 (**). The same control cohort was used for the experiments in Supplementary Figures 1, 2. [file Image2.tif]
